# Une rare triple association de panniculite mésentérique, appendicite aigüe et syndrome de Koenig chez un patient opéré pour une occlusion intestinale aigüe fébrile: à propos d’un cas

**DOI:** 10.11604/pamj.2023.45.57.19448

**Published:** 2023-05-24

**Authors:** Manix Ilunga Banza, Trésor Kibangula Kasanga, Augustin Kibonge Mukakala, Dimitri Kanyanda Nafatalewa, Eddy Wasso Milinganyo, Wolf Lisasi, Emmy Manda Kisimba, Mylord Kambu Ngoma, Serges Ngoie Yumba, Yannick Tietie Ben N'dwala

**Affiliations:** 1 Département de Chirurgie, Cliniques Universitaires de Lubumbashi, Lubumbashi, République Démocratique du Congo,; 2 Département de Réanimation-Anesthésie, Cliniques Universitaires de Lubumbashi, Université de Lubumbashi, Haut Katanga, République Démocratique du Congo

**Keywords:** Panniculite mésentérique, appendicite, syndrome de Kœnig, cas clinique, Mesenteric panniculitis, appendicitis, König´s syndrome, case report

## Abstract

La panniculite mésentérique est une affection primitive inflammatoire du mésentère associant de façon variable des lésions de nécrose, d´inflammation et de fibrose du tissu graisseux. Elle peut être idiopathique (primitive) ou secondaire (associée) à d´autres pathologies, asymptomatique et de découverte fortuite ou par une douleur abdominale ou des complications (occlusion intestinale ou une péritonite). Nous présentons le cas d´un patient de 53 ans, reçu pour douleur abdominale aiguë, arrêt de matières et de gaz dans un contexte fébrile. Aux antécédents, des douleurs abdominales chroniques sous forme d´un syndrome de Koenig avait été noté et des épigastralgies depuis plusieurs années. A l´examen physique, l´état général marqué par un faciès souffrant et l´examen abdominal avait noté un léger ballonnement, une sensibilité marquée dans la fosse iliaque droite (FID) et en péri-ombilical, sans défense ni contracture et des borborygmes à l´auscultation avec toucher rectal normal. Un diagnostic d´occlusion intestinale fébrile avait été retenu avec suspicion d´une appendicite mésocoeliaque. La radiographie de l'abdomen à blanc et l´échographie ont permis de confirmer le diagnostic d´occlusion abdominale. Au décours de la laparotomie exploratrice, une sténose fonctionnelle iléale (syndrome de Koenig) découvert à 1m20 de la jonction iléo-coecale; cette dernière étant le siège de multiples adhérences faisant découvrir à l´adhésiolyse un appendice hyperhémié, long de 15cm dont l´examen anatomopathologique révèle une muqueuse siège d´infiltrat inflammatoire et une paroi riche en polynucléaires; une infiltration du mésentère iléal sous forme de modification de coloration (rougeâtre et grisâtre par endroit) et de petites nodosités avec friabilité et déchirure à la simple manipulation a fait suspecter le diagnostic de panniculite mésentérique confirmée par les examens anatomopathologiques montrant une réaction inflammatoire dans le tissu graisseux prélevé avec infiltration par des macrophages, associée à des lésions de nécrose en plage et dégénérescence. Le traitement avait consisté en une vidange intestinale, une appendicectomie antérograde, et une association corticoïde (Dexamethasone 24 mg/jour) et chymotrypsine (10000 UI/jour). L´évolution était bonne et une sortie du patient au 10^e^ jour post-opératoire avec un suivi clinique et paraclinique pendant 3 mois à la recherche d´une autre pathologie associée méconnue ou pouvant survenir précocement.

## Abstract (en)

Mesenteric panniculitis is a primary inflammation of the mesentery with variable necrosis, inflammation and fibrosis of the fatty tissue. It can be idiopathic (primary) or secondary (associated) to other diseases, asymptomatic and accidentally discovered or revealed by abdominal pain or complications (intestinal obstruction or peritonitis). We here report the case of a 53-year-old patient, admitted with acute abdominal pain, cessation of the transit of materials and gases, in a febrile context. Patient’s history included chronic abdominal pain suggesting König´s syndrome and epigastralgia lasting several years. Physical examination showed sore face and abdominal examination revealed mild bloating, marked tenderness in the right iliac fossa (RIF) and in the periumbilical region, without guarding or rigidity and borygms heard on auscultation and with normal rectal examination. A diagnosis of bowel obstruction and fever was made, with suspicion of meso-celiac appendicitis. Abdominal X-ray without preparation and ultrasound confirmed the diagnosis of bowel occlusion. Exploratory laparotomy revealed functional stenosis of the ileum (König´s syndrome) at 1.20m from the ileocecal junction, with multiple adhesions. Adhesiolysis revealed hyperemic appendix measuring 15cm long, whose anatomo pathological examination showed a mucous membrane with inflammatory infiltrate and a wall rich in polynuclear cells. Infiltration of the ileal mesentery causing color change (reddish and greyish in some areas) and small nodosities with friability and tearing on simple handling led to suspicion of mesenteric panniculitis, then confirmed by anatomopathological examination, showing inflammatory reaction in the fatty tissue specimen with infiltration by macrophages, associated with necrotic patches and degeneration. Treatment was based on bowel emptying, anterograde appendectomy, and a combination of corticosteroid (Dexamethasone 24 mg/day) and chymotrypsin (10000 IU/day). The patient´s outcome was good and he was discharged in the 10^th^ postoperative day. The patient underwent clinical and paraclinical follow-up (3 months) for another unknown associated pathology or a pathology that may have occurred early.

## Introduction

Une occlusion intestinale aigüe est un arrêt partiel ou total du transit intestinal caractérisée par une douleur abdominale, un arrêt de matières et surtout des gaz, des vomissements et un météorisme abdominal. Elle peut être mécanique ou fonctionnelle. L´occlusion mécanique relève de la chirurgie alors que celle fonctionnelle n'est à priori pas chirurgicale sauf si elle est secondaire à un foyer intra-abdominal infectieux tel une péritonite localisée sur appendicite suppurée. La présence de la fièvre fait évoquer une occlusion fébrile qui peut traduire une sigmoïdite, une pancréatite, une appendicite surtout celle de topographie méso-cœliaque, une diverticulite de Meckel. Le syndrome de Koenig est dû à un dysfonctionnement de l´intestin grêle cependant il peut être un mode révélateur d´une pathologie tumorale de l´intestin grêle [1]. Il est caractérisé lors de la crise par une distension abdominale car le volume de l´intestin en amont de l´obstacle augmente, un hyperpéristaltisme, épisodes d´alternance diarrhée et constipation et des coliques abdominales violentes empêchant le patient de rester à la verticale. Les signes associés sont nombreux (météorisme, borborygmes, arrêt des gaz et résolution brutale avec résolution à type de débâcle aéro-hydrique; ces symptômes sont en rapport avec un mécanisme occlusif transitoire du tube digestif.

Le mésentère peut être le siège de plusieurs pathologies aussi bien tumorales qu´inflammatoires avec un retentissement sur l´intestin. C´est le cas de la panniculite mésentérique qui est une affection primitive inflammatoire du mésentère associant de façon variable des lésions de nécrose, d´inflammation et de fibrose du tissu graisseux [2]. En fonction du contingent dominant, on distingue respectivement la mésentérique lipodystrophique, la panniculite mésentérique et la mésentérite rétractile [1]. Des épisodes d´occlusion intestinale peuvent être le mode révélateur d´une panniculite mésentérique [2]. La prévalence de la panniculite mésentérique rapporté par la littérature varie de 0,16 à 0,6% mais son incidence serait en nette augmentation ces dernières années et ceci en rapport avec l´amélioration des moyens d´imagerie médicale [3]. La physiopathologie de la panniculite mésentérique est mal connue; l´hypothèse d´une réaction inflammatoire du tissu graisseux mésentérique en réponse à une pathologie pré-existante ou co-existante par le biais d´une dégradation enzymatique excessive bien que les associations avec des pathologies inflammatoires et néoplasiques en particulier lymphomateuses auraient été décrites [2]. Elle peut être idiopathique ou primitive sans aucune autre pathologie associée mais aussi secondaire qui serait une panniculite d´accompagnement. Il convient donc de rechercher une pathologie infectieuse, néoplasique ou inflammatoire sous-jacente [2,3] sachant qu´aucun lien de causalité n´a jamais été établi [4].

Le mode de révélation est très variable. La panniculite mésentérique est souvent asymptomatique et de découverte fortuite lors d´un *computed tomography scan* abdominal réalisé pour autre cause [1,2] mais la douleur abdominale reste le signe le plus fréquent [1], parfois une masse est palpée accompagnée d´autres signes tels que la fièvre, l´altération de l´état général, diarrhée, iléus, signes systémiques (arthralgie, érythème noueux) [5]. Des biopsies chirurgicales au cours des laparotomies ou laparoscopies permettent de poser le diagnostic et certains auteurs affirment que seul l´examen histologique peut la confirmer et peut mettre en évidence un diagnostic différentiel simulant une panniculite [5]. Sur le plan anatomopathologique [1], les lésions sont reparties en trois à savoir la lipodystrophie mésentérique caractérisée par des lésions de dégénérescence puis de nécrose des adipocytes en plage, avec des zones mal limitées constituées de fibroblastes entourant des plages résiduelles de tissus graisseux normal. La panniculite mésentérique est caractérisée par des lésions inflammatoires avec infiltrat du mésentère par des agrégats des macrophages phagocytant les lipides (lipophages) avec parfois un infiltrat de cellules géantes, et un degré variable de fibrose. La mésentérite rétractile est caractérisée par une fibrose massive constituée de collagène et de fibroblastes entrainant un épaississement et une déformation du mésentère [6].

L´évolution de la panniculite mésentérique est le plus souvent spontanément favorable avec résolution complète de la symptomatologie dans un délai variable mais la majorité d´étude est unanime sur l´efficacité des corticoïdes [3] tandis que le traitement chirurgical est réservé aux complications obstructives digestives ou vasculaires et consiste généralement en une adhésiolyse, exérèse partielle ou complète de la masse mésentérique [6], la réalisation d´un geste de dérivation ou résection intestinale segmentaire [2]. Le but de cette publication est d´une part de présenter cette triple association d´une panniculite mésentérique, d´une appendicite et d´un syndrome de Koenig chez un patient opéré pour occlusion intestinale fébrile non encore décrit dans la littérature, et d´autre part de présenter les éventualités de diagnostic et de traitement.

## Patient et observation

**Présentation du patient:** il s´est agi d´un patient âgé de 53 ans, venu consulter au Centre Hospitalo-universitaire de la SNCC en date du 24 janvier 2019 pour douleur abdominale ainsi que l´arrêt des matières et des gaz. L´histoire de la maladie remontait à 24 heures de notre consultation par la survenue brutale d´une douleur abdominale suivie quelques heures après d´une impossibilité d´émission des gaz pour laquelle le patient s´était automédiqué au Spasta (antispasmodique) comprimé de 10 mg sans succès. La persistance de l´impossibilité d´émission des gaz, de la douleur abdominale et son exacerbation, la sensation d´une distension abdominale avaient motivé le patient à consulter au Complexe Hospitalo-universitaire de SNCC pour une prise en charge. Aucun antécédent chirurgical n´a été noté, notion d´épigastralgie depuis plusieurs années, prise régulière d´alcool depuis plusieurs années mais pas de tabac. Au complément d´anamnèse, le patient avait signalé une douleur abdominale de survenue brutale devenant de plus en plus intense, paroxystique, localisée dans la région ombilicale et la fosse iliaque droite, vomissement liquidien une seule fois, une fièvre depuis 4 jours, arrêt de matières et de gaz avec sensation de gargouillement.

**Renseignements cliniques:** à l´examen physique, l´état général était marqué par un faciès souffrant. Les conjonctives palpébrales étaient colorées, les bulbaires anictériques, la bouche était propre et la langue était humide, aucune adénopathie n´avait été retrouvée à la palpation. Le thorax était eupnéique à 24 cycles par minutes, cœur normocarde à 95 battements par minute. L´abdomen était légèrement ballonné, la respiration abdominale présente, l´ombilic n´était pas déplissé, pas de voussure visible ni de circulation collatérale. La palpation superficielle ne décelait pas une hyperesthésie cutanée. La palpation profonde a décelé une douleur abdominale surtout marquée dans la région de la fosse iliaque droite et péri-ombilicale sans empâtement, ni défense, ni contracture. Le cri ombilical était absent. Nous n´avions pas noté de matité déclive mobilisable. La matité pré-hépatique était conservée. A l´auscultation, nous avions noté une exacerbation des bruits hydro-aériques marquée dans la région droite de l´abdomen. Au toucher rectal, le sphincter était tonique, l'ampoule rectale vide, la muqueuse rectale souple, la prostate de volume normal, non sensible avec un sillon médian présent, pas de masse intra-rectal perçue, le cul de sac de Douglas non bombé et non sensible.

**Démarche diagnostique:** un diagnostic clinique d´occlusion intestinale fébrile avait été retenu avec comme entité nosologique une appendicite mésocoeliaque, une diverticulite de Meckel, un abcès profond intra-abdominale à exclure. Nous avions ainsi demandé des examens paracliniques faits de: 1) la radiographie abdomen à blanc patient débout avec visualisation des hémicoupoles diaphragmatiques qui a révélée l´absence de pneumopéritoine (pas de croissant gazeux sous diaphragmatique) mais la présence de 2 gros niveaux hydro-aériques centraux et d´un autre petit central tous plus larges que hautes suggestif d´une occlusion de l'intestin-grêle. 2) L´échographie abdomino-pelvienne avait montré un foie et une vésicule biliaire intacte, un pancréas et une rate normale, des reins normaux, aucune masse intra-abdominale, appendice non visualisée à l´échographie. Les anses intestinales sont fortement dilatées avec rétention d´eau et de gaz sans aucune suspicion de lésions mésentériques. Un diagnostic d´occlusion intestinale avait été retenu par l´échographe. 3) Les résultats de laboratoire avant l´intervention chirurgicale que voici: A) bactériologie: Widal TH (-) TO (-); sérologie HIV: négative; B) biochimie: glycémie 88 ml/dl; protéine C-réactive (CRP): 65,2 mg%; urée 24mg/dl; créatinine 1,12mg/dl; sodium 138mEq/l, potassium 4,3mEq/l; calcium 8,5mg/dl; C) h ématologie: érythrocytes 448000; hématocrite 35%; hémoglobine 16g/dl; hématocrite 49%, vitesse de sédimentation 56mm/h; plaquette 255000/mm^3^, globules blancs 112000/mm^3^, volume globulaire moyen 89,2µm^3^, formule leucocytaire granulocytes 45%, lymphocytaire 47%, monocytes 8%. D) Goutte épaisse négative (0 parasites/µl). E) Temps de saignement 2'00", temps de coagulation 4'30", groupe sanguin et rhésus 0^+^. Ainsi, après un examen clinique et paraclinique une occlusion intestinale fébrile avait donc été retenue et une décision d´opérer par une laparotomie exploratrice avait été prise après une évaluation anesthésique.

**Figure 1:**
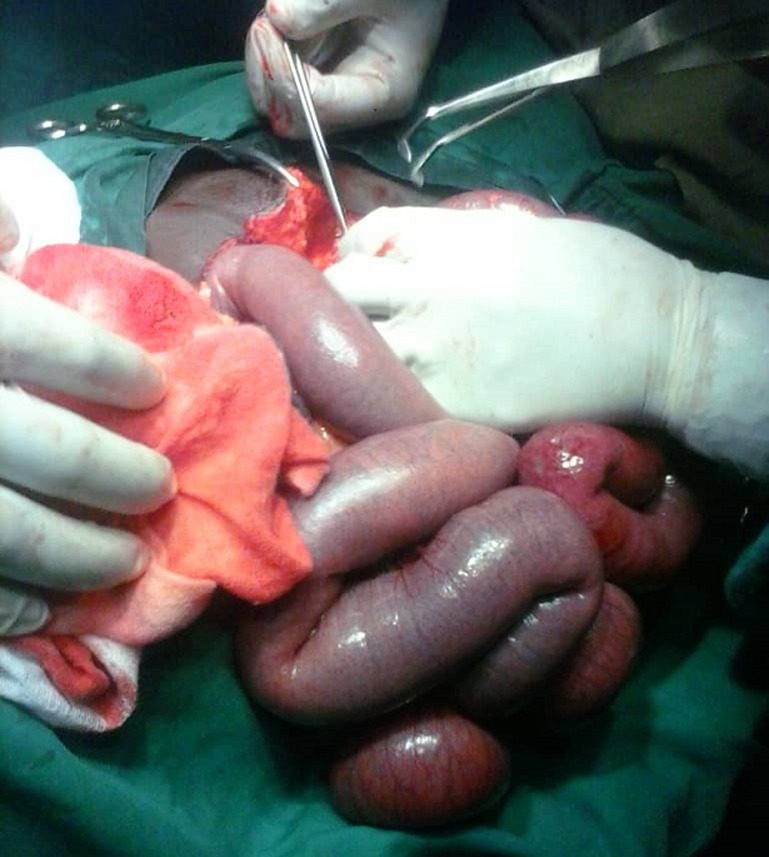
dilatation importante des anses en amont de la zone sténotique

**Figure 2:**
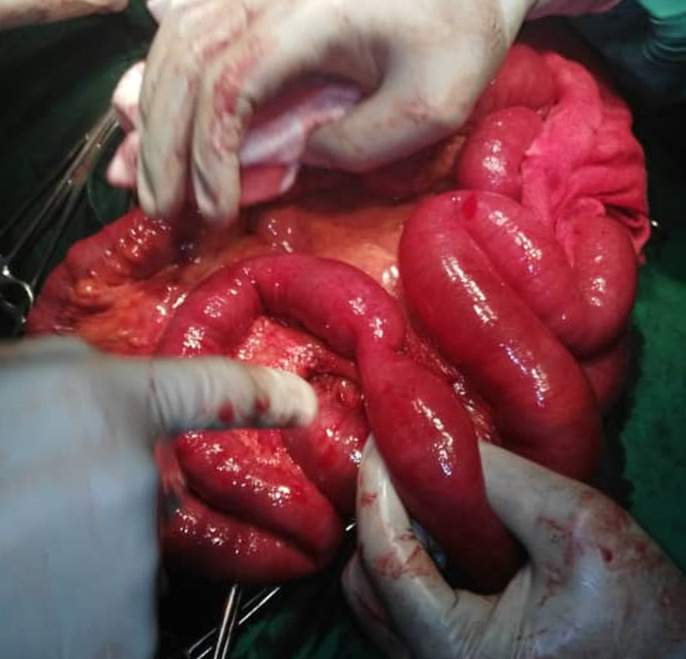
zone sténotique siège du syndrome de Koenig; obstacle fonctionnelle

**Intervention thérapeutique:** notre patient était classé ASA III, et une réanimation pendant 8 heures avait été instaurée. Elle a consisté en: la pose d´une sonde nasogastrique numéro 16, la pose d´une sonde vésicale à demeure numéro 18, la pose d´une voie veineuse centrale avec une réanimation hydrique selon la formule de Lewis repartie en sérum physiologique un litre pendant 3 heures, hémacèle un litre pendant 2 heures et Ringer Lactate un litre pendant 3 heures. Une antibiobioprophylaxie au Ceftriaxone avait été instaurée 2 grammes avant l´intervention chirurgicale. La laparotomie médiane sus et sous-ombilicale a été la voie d´abord. A l´entrée dans la cavité abdominale, présence d´un liquide de transsudation d´aspect clair sans odeur particulière. Une dilatation importante des anses grêles (Figure 1) avec présence à 1m20cm d´une zone rétrécie séparant les anses plates en avale siège du syndrôme de Koenig (Figure 2). Cette zone rétrécie a la même consistance que le reste des anses et ne possède donc pas d´obstacle organique car la perméabilité de la lumière intestinale avait été vérifiée manuellement par la pince digitale supérieure pouce-index. La chiquenaude exercée sur les anses démontre un péristaltisme venant butter contre cette région sténotique mais le refoulement manuel du contenu intestinal d´amont franchit cette zone aux pris d´un borborygme.

**Figure 3:**
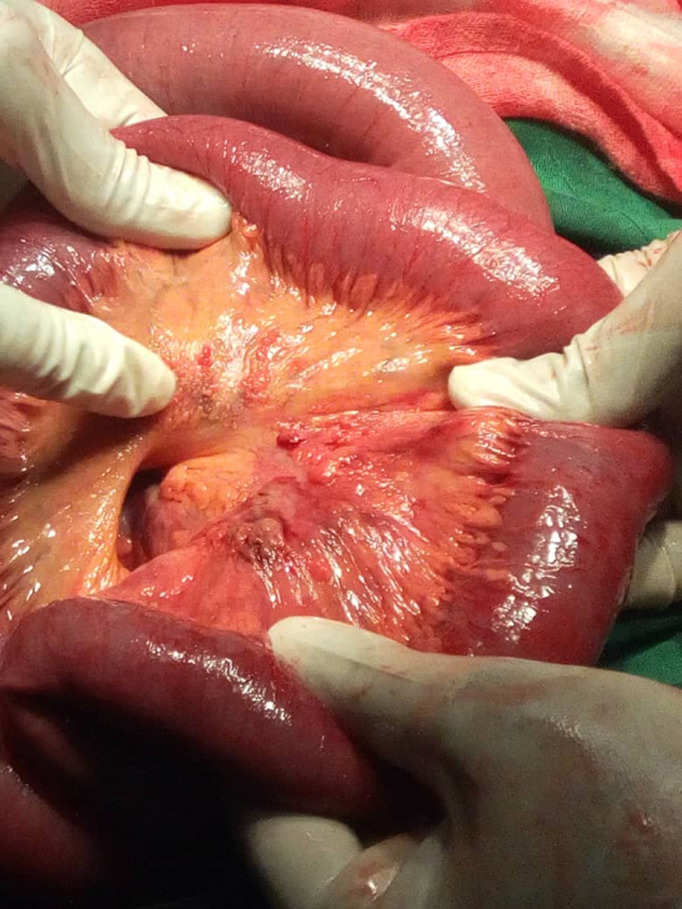
coloration anormale du mésentère iléal avec petites nodosités infiltrant le mésentère devenu friable

**Figure 4:**
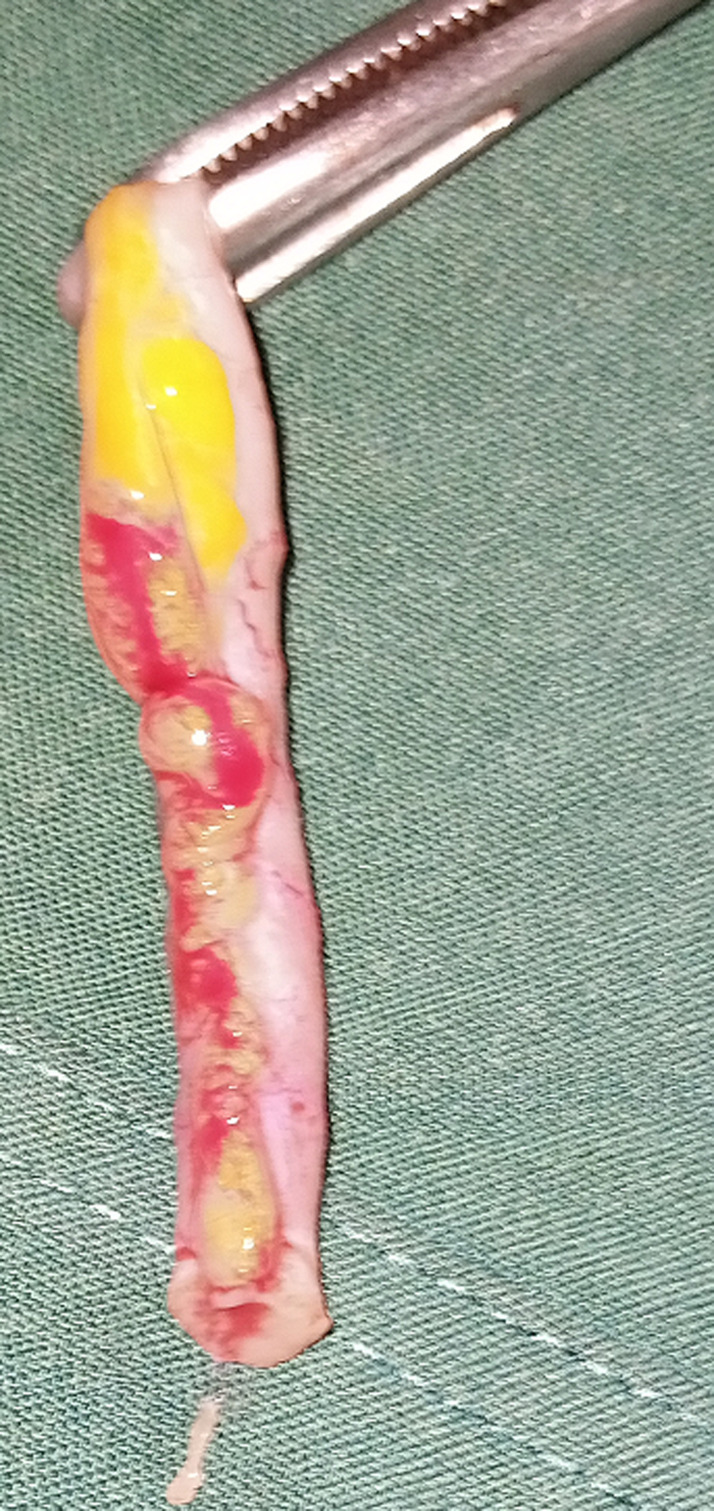
appendice inflammé enlevé au cours de l'intervention réalisée

Au niveau du mésentère iléal la présence d´une coloration anormale rougeâtre et grisâtre par endroit avec infiltration des petites nodosités infra-centimétriques parsemées dans le mésentère devenu friable (Figure 3) mais sans aucune anomalie des anses grêles en regard qui sont bien rosées. La moindre manipulation de cette région mésentérique entraine sa déchirure témoignant de sa fragilité par rapport au reste du mésentère. Aucune adénopathie mésentérique n´a été mise en évidence. Nous avions ainsi réalisé un prélèvement du mésentère infiltré pour un examen anatomopathologique. Au niveau de la jonction iléo-coecale, on note la présence de beaucoup d´adhérences englobant l´appendice dont la base n´était plus visible, le cœcum et les 15 derniers centimètres de l´iléon terminal pris dans ces tissus fibreux adhérentiels. La prise en charge chirurgicale a consisté en une adhésiolyse de la région iléo-coecale; ce qui a permis de mettre en évidence un appendice hyperhemié avec une richesse en vascularisation visible sur sa séreuse, long de 15 cm, avec son méso intacte. Nous avions donc ainsi réalisé une appendicectomie antérograde (Figure 4) et apporté la pièce en anatomopathologie pour des examens histologiques. Nous avons procédé à la vidange du contenu intestinale par l´anus en partant de l´angle de Treitz nous permettant ainsi non seulement de vérifier le franchissement de la sténotique mais aussi de rechercher une tumeur du grêle ou colique qui n´étaient pas présentes chez notre patient. La région sténotique intestinale franchit facilement lors de la vidange n´a pas été reséquée car aucun obstacle mécanique. Le nettoyage de la cavité abdominale avec du sérum physiologique tiède. La cavité abdominale a été ensuite refermée en deux plans au vicryl.

**Suivi et résultats des interventions thérapeutiques:** le patient avait séjourné 72 heures en réanimation. Une antibiothérapie faite de Céfotaxime un gramme toutes les 8 heures en intraveineuse directe et une association de chymotrypsine flacon de 5000UI à raison de 2 fois un flacon par jour et la corticothérapie faite de la dexaméthasone 3 x 8 mg par jour avaient été instaurée. Le retour de transit était effectif après 48 heures avec une disparition de la douleur abdominale, une nette diminution des sécrétions gastriques revenue à la normale et une émission des selles et des gaz. L´abdomen non ballonné, souple, dépressible, la sensibilité uniquement péri-lésionnelle. Le premier pansement a été réalisé au 5^e^ jour post-opératoire montrant un pansement médian propre et sec. Le malade a été libéré de l´hôpital au 10^e^ jour post-opératoire après une ablation complète des fils de suture sans aucun signe d´infection locale de la plaie opératoire.

Les analyses anatomopathologiques avaient trouvé comme résultats: 1) le tissu prélevé a montré la présence des adipocytes présentant des lésions de nécrose en plage, avec des lésions de dégénérescence en leur sein, d´autres sont le siège d´une réaction inflammatoire avec infiltration par des agrégats de macrophages. 2) Un diagnostic de lésion de dystrophie et d´une réaction inflammatoire au sein de ce tissu graisseux évoque le syndrôme de Christian-weber. 3) Au niveau de l´appendice, macroscopiquement dilaté, congestif et recouvert de fausses membranes et microscopiquement, ulcération plus ou moins profonde de la muqueuse avec infiltrat inflammatoire de toute la paroi avec richesse en polynucléaires neutrophiles évoquant une appendicite aigüe. Le patient a ainsi été suivi pendant 3 mois à la recherche d´une autre pathologie associée et exclure une quelconque tumeur pouvant survenir ou passée inaperçue. Cliniquement, le patient n´a présenté aucun problème et sur le plan paraclinique, deux radiographies thoraciques et deux échographies abdominopelviennes ont été réalisées à intervalle d’un mois; aucune autre pathologie associée n´a été trouvée chez notre patient.

**Consentement éclairé:** après des explications claires et détaillées, pour la publication de ce travail, nous avons obtenu du patient par écrit son consentement éclairé.

## Discussion

Une panniculite mésentérique peut être idiopathique ou primitive sans aucune autre pathologie associée mais aussi secondaire qui serait une panniculite d´accompagnement. Il convient donc de rechercher une pathologie infectieuse, néoplasique ou inflammatoire sous-jacente [2,3] sachant qu´aucun lien de causalité n´a jamais été établi [6]. Dans notre observation, le patient présentait une appendicite. Confirmant diverses littératures qui dit que la panniculite mésentérique est souvent associée soit à un cancer situé à distance soit à des affections bénignes abdomino-pelviennes ou rétro-péritonéales [6]. C´est une pathologie qui touche les individus de la cinquantaine avec un sexe ratio 2/1 en faveur des hommes, ce qui corrobore avec l´âge de 53 ans bien que l´âge médian étant de 60 ans [2] et le sexe masculin de notre patient, quoique quelques exceptionnels cas pédiatriques soient publiés [6]. Le site le plus touché est le mésentère (85% des cas), l´atteinte du mésocolon (mésosigmoide essentiellement) étant beaucoup plus rare (10%) [6] corroborant avec la localisation observée chez notre patient sur le mésentère iléal alors que le mésocolon était intact.

Cette pathologie peut être asymptomatique dans 30 à 50% des cas avec une symptomatologie abdominale absente dans 30% des cas. Cependant dans la série d´Emory *et al*. les signes rapportés les plus fréquents étaient une douleur abdominale (34%), une masse abdominale (30,8% à 50%) et un syndrome occlusif (30,8%) [3]. Dans la série de Vuitton [6], les signes retrouvés sont: douleur abdominale, fièvre, trouble de transit ou des vomissements. Notre observation rejoint donc les divers éléments cliniques rapportés par la littérature car notre patient cliniquement avait présenté un trouble de transit, un syndrome de Koenig, la fièvre. Cependant certains autres patients peuvent présenter en dehors des signes ci-hauts cités d´autres signes systémiques (arthralgie, érythème noueux) et parfois même des nodosités sous cutanées érythémateuses [3,4].

Le bilan hématologique peut témoigner d´un processus inflammatoire d´intensité variable tels (une vitesse de sédimentation à 3 chiffres (105 mm à la 1^re^ heure) et une protéine C réactive très élevée (166 mg) [4] ce qui corrobore avec les résultats de notre patient qui a présenté une vitesse de sédimentation de 56 mm à la 1^re^ heure et une protéine C réactive à 65mg.

Le fait que la panniculite mésentérique soit souvent associée à d´autres pathologies comme rapportées par diverses littératures telles que néoplasiques (lymphomes, adénocarcinomes), infections bactériennes (tuberculose), traumatisme ou chirurgie abdominale, une prise de toxique (médicament), une radiographie (radiothérapie), allergique, une ischémie mésentérique, une maladie auto-immune, un rejet de greffe au cours d´une transplantation d´intestin grêle [2-5]; il convient de faire des investigations paracliniques poussées pour déterminer s'il s´agit d´une forme primitive ou secondaire. C´est ainsi que chez notre patient en post-opératoire, une nouvelle échographie et un abdomen à blanc avaient été réalisés, un bilan inflammatoire et une intradermoréaction à la tuberculine avaient également été réalisés du patient pour exclure une pathologie méconnue.

Plus que l´échographie, c´est la tomodensitométrie et l´imagerie par résonnance magnétique qui permettent d´évoquer le diagnostic et de faire les biopsies [4] mais notre patient venu dans un tableau d´occlusion intestinale explique l´impossibilité de réaliser ces examens pour poser le diagnostic. Le scanner abdominal avec injection du produit de contraste permet d´étayer le diagnostic positif de panniculite mésentérique, de guider une éventuelle biopsie percutanée et de surveiller de façon non invasive les lésions [7]; il montrera une augmentation de la densité de la graisse mésentérique « misty mesentery » [6] associée à des nodules tissulaires au sein de cette infiltration. Dans notre série la découverte était faite au décours d´une laparotomie exploratrice indiquée pour une occlusion intestinale fébrile.

Des biopsies chirurgicales au cours des laparotomies ou laparoscopies permettent également de poser le diagnostic de panniculite mésentérique avec parfois de possibilités pour des biopsies échoguidées. Hakguder *et al*. ont défini trois formes de lésions de panniculite mésentérique en se fondant sur des caractéristiques per opératoires [4] à savoir type I (infiltration et épaississement diffus du mésentère), type II (la forme nodulaire unique pseudotumorale) et type II (la forme multinodulaire). Dans la série de Hakguder *et al*. [4], les lésions les plus fréquentes étaient celles de type I (42%), suivie de type II (32%) et enfin celles de type III (26%) alors que dans celle d´Emory *et al*. [3], plus de lésions de type II (69%) contre 18% de type III et 13% de type I. Au regard de cette classification, notre patient présentait un type II marqué par de petits nodules millimétriques occupant une vaste portion du mésentère iléal.

Sur le plan anatomopathologique, les lésions sont reparties en trois à savoir: la lipodystrophie mésentérique caractérisée par des lésions de dégénérescence puis de nécrose des adipocytes en plage, avec des zones mal limitées constituées de fibroblastes entourant des plages résiduelles de tissus graisseux normal; la panniculite mésentérique caractérisée par des lésions inflammatoires avec infiltrat du mésentère par des agrégats des macrophages phagocytant les lipides (lipophages) avec parfois un infiltrat de cellules géantes, et un degré variable de fibrose; la mésentérite rétractile caractérisée par une fibrose massive constituée de collagène et de fibroblastes entrainant un épaississement et une déformation du mésentère. Les résultats anatomopathologiques de notre patient avaient montré les signes histologiques compatibles avec une panniculite mésentérique.

Le traitement de la panniculite mésentérique ne fait pas l´objet d´un consensus et ne s´adresse qu´aux formes symptomatiques. Tous les auteurs s´accordent au traitement médical d´emblée et le traitement chirurgical n´est réservé qu´aux complications car les lésions de panniculite sont le plus souvent non résécables [3,6]. L´unanimité est faite quant à l´usage des corticoïdes mais quant aux médicaments à y adjoindre plusieurs divergences ressortent des littératures c´est notamment des anti-inflammatoires ou immunosuppresseurs pour certains, le tamoxifène, la progestérone, la colchicine (20mg/j) [6]. Notre patient a ainsi été mis sous une association d´un corticoïde (prednisolone) associée à un anti-inflammatoire (Chymotrypsine).

Le traitement chirurgical est envisagé pour une visée diagnostique, sous laparoscopie ou laparotomie, pour la réalisation des biopsies [8] et en urgence pour traiter les complications digestives justifiant ainsi la prise en charge chirurgicale chez notre patient qui a présenté une complication chirurgicale l´occlusion intestinale; ce qui nous a permis non seulement de poser le diagnostic de panniculite mésentérique en réalisant un prélèvement biopsique du mésentère infiltré, mais aussi de trouver la pathologie associée qui était une appendicite et de la traiter sans oublier la mise en évidence du syndrome de Koenig.

Parfois les gestes chirurgicaux peuvent être une dérivation intestinale interne (by-pass), ou externe (stomie) parfois même une résection intestinale segmentaire avec double stomie en cas d´ischémie mésentérique [9]; notre patient n´ayant pas présenté de telles complication justifie notre acte chirurgical beaucoup moins mutilante. Notre acte chirurgical corrobore avec la littérature qui affirme qu´en dehors des explorations diagnostiques, le recours à la chirurgie est rare en cas d´atteinte mésentérique, elle n´est même pas justifiée car l´évolution des lésions est habituellement favorable même en l´absence de traitement avec restitution *ad integrum*, dans un degré variable de plusieurs mois à plusieurs années (jusqu´à 11 ans) [9].

La surveillance de l´efficacité du traitement doit donc rester avant tout clinique et biologique au point où certaines littératures recommandent un *computerized tomography scan* abdominal de contrôle à distance (un fois par an) serait néanmoins souhaitable afin de vérifier l´absence d´une pathologie tumorale évolutive justifiant de ce fait notre surveillance clinique et paraclinique durant les 3 mois post-opératoires par des échographies abdominales, radiographies thoraciques, une endoscopie digestive haute et basse et un toucher rectal. Aucune anomalie apparue dans les 3 mois de surveillance dans notre observation.

## Conclusion

La panniculite mésentérique, entité rare et non encore publiée dans notre milieu reste une pathologie à connaitre. La recherche d´une pathologie sous-jacente est à exclure impérativement.
